# Impact of Storage Conditions on Bronchoalveolar Lavage Fluid Analysis: A Human Study

**DOI:** 10.3390/diagnostics15111386

**Published:** 2025-05-30

**Authors:** Yu Shionoya, Kanae Maruyama, Takeshi Kawasaki, Mayumi Ono, Yushi Murai, Ryutaro Hirama, Dai Horiuchi, Noriko Sakuma, Shinsuke Kitahara, Shun Sato, Kohei Takahashi, Yoshihito Ozawa, Takuji Suzuki

**Affiliations:** 1 Department of Respirology, Graduate School of Medicine, Chiba University, 1-8-1 Inohana, Chuo-ku, Chiba City 260-8670, Japan; 2 Biostatistics Section, Clinical Research Center, Chiba University Hospital, 1-8-1 Inohana, Chuo-ku, Chiba City 260-8677, Japan

**Keywords:** bronchoalveolar lavage, total cell count, differential cell count, interstitial lung disease, neutrophils

## Abstract

**Background**: Bronchoalveolar lavage fluid (BALF) analysis is essential for the accurate diagnosis and management of interstitial lung diseases (ILDs). Despite established guidelines, variability in sample handling may affect diagnostic accuracy. This study aimed to evaluate how different storage conditions impact BALF cell counts and differentials to guide optimal sample handling practices. **Methods**: Forty patients who underwent BAL at Chiba University Hospital from June to December 2024 were included. BALF samples were allocated into five groups based on processing conditions: immediate analysis within 1 h, storage at either at 4 °C or room temperature (RT) for 6 h, or storage at 4 °C or RT for 24 h. Total cell counts (TCC) and differential counts were measured and compared among conditions. **Results**: TCC remained stable over 24 h at both 4 °C (*p* = 0.86) and RT (*p* = 0.90). Similarly, the percentages of eosinophils, lymphocytes, and macrophages did not significantly change at either temperature (all *p* > 0.05). Notably, neutrophil percentages showed a significant decline over time under both storage conditions—at 4 °C (*p* = 0.02) and at room temperature (*p* < 0.01). Post hoc tests revealed a notable decreasing trend at 6 h and significant reductions by 24 h at 4 °C (*p* = 0.09 and *p* = 0.02, respectively), and significant decreases at both 6 and 24 h at RT (*p* = 0.01, <0.01). **Conclusions**: Among the various cell types in BALF, neutrophil proportions are particularly susceptible to storage conditions, showing a significant decline over time—especially at room temperature—while other cell types remain stable for up to 24 h. Therefore, prompt processing or appropriate refrigeration of BALF is essential to ensure reliable cytological analysis and accurate clinical interpretation.

## 1. Introduction

Bronchoalveolar lavage (BAL) has been employed as a diagnostic tool for evaluating interstitial lung diseases (ILDs) since the 1970s [1]. It involves instilling and retrieving normal saline to collect secretions from the apical surfaces of the bronchial and alveolar epithelia in patients with ILDs [2]. Clinical guidelines published in 2012 concluded that while BAL fluid (BALF) analysis may support ILD diagnosis, it lacks prognostic value and does not reliably predict therapeutic response [2]. However, subsequent studies have suggested that BALF analysis can offer prognostic information and help predict treatment outcomes [3,4,5]. More recent updates to ILD guidelines further support a potential diagnostic role for BALF analysis in conditions such as idiopathic pulmonary fibrosis (IPF) and hypersensitivity pneumonitis (HP) [6,7]. These findings underscore the continued relevance of BALF analysis in the clinical management of ILDs.

Clinical guidelines recommend prompt processing of BALF samples to ensure optimal diagnostic accuracy [2,8]. Nevertheless, the effect of BALF storage conditions on analytical outcomes remains insufficiently studied [9,10]. Furthermore, despite these recommendations, significant inter-institutional variability in BAL processing has been observed, particularly in Japan, with differences in time from BALF retrieval to analysis and processing locations. Notably, approximately 20% of facilities outsource BALF analysis instead of performing it in-house, and around 10% report that respiratory physicians conduct the analysis. In some cases, the time to analysis is not tracked at all [8,11]. Such variability can compromise diagnostic accuracy, potentially leading to misdiagnosis and inappropriate management of patients with ILDs. Hence, standardizing BAL procedures is urgently needed. To address this knowledge gap, the present study investigated the impact of storage conditions, specifically temperature and duration, on BALF cellular analysis. We hypothesized that delayed processing and inadequate storage conditions would adversely affect BALF cytological outcomes. Clarifying these effects could inform standardized processing protocols, thereby improving diagnostic accuracy and patient management in ILDs. Our findings underscore the necessity for immediate BALF analysis or standardized storage practices to preserve diagnostic integrity.

## 2. Materials and Methods

### 2.1. Study Design and Participants

This prospective, single-center observational study included consecutive adult patients who underwent BAL for diffuse lung diseases at Chiba University Hospital between June and December 2024. Patients with suspected definitive etiologies of pneumonia, including drug-induced pneumonitis, radiation-induced pneumonitis, pulmonary infection, were excluded. Additionally, patients with insufficient BALF recovery volume for analysis and those who withdrew written informed consent were also excluded.

The study protocol was approved by the Ethical Review Board of the Graduate School of Medicine, Chiba University (approval number 2083) and was conducted in accordance with the ethical principles of the Declaration of Helsinki (1984) and its subsequent amendments. Written informed consent was obtained from all participants.

In this study, generative artificial intelligence was not utilized in the creation or analysis of data.

### 2.2. BAL Procedure

BAL was performed in accordance with the American Thoracic Society (ATS) and Japanese Clinical Guidelines [2,8]. The target lung segment was selected based on high-resolution computed tomography (HRCT) performed prior to bronchoscopy. A fiberoptic bronchoscope (BF-1TQ290; Olympus, Tokyo, Japan) was positioned within the selected segment. A total of 150 mL of normal sterile saline was instilled in three separate 50 mL aliquots. Following the instillation of each aliquot, the fluid was retrieved using gentle negative suction, maintaining a pressure of less than 100 mmHg.

### 2.3. BALF Handling, Processing, and Analysis

**Figure 1:**
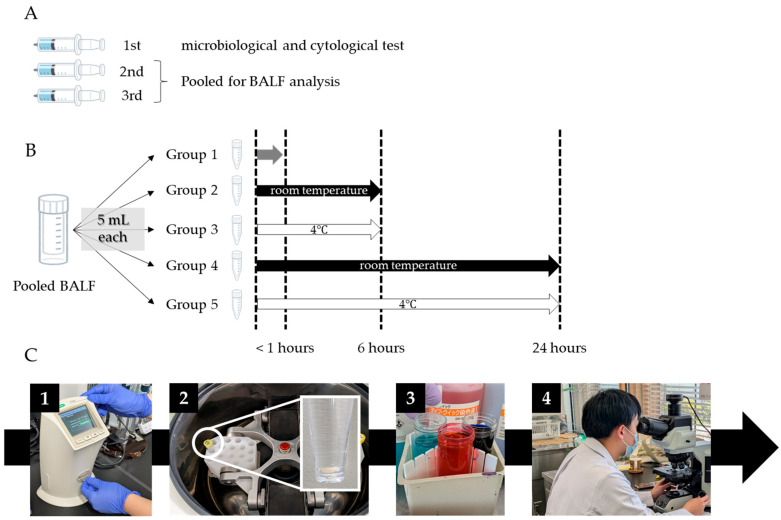
Procedures for BALF handling, processing, and analysis. (**A**). The first recovered aliquot is used for microbiological and cytological testing, while the second and third aliquots are pooled for further BALF analysis. (**B**). The pooled samples are divided into five 5 mL aliquots and stored under the designated conditions. (**C-1**). Total cell count is measured using the automated cell counter. (**C-2**,**C-3**). The samples are centrifuged at 3000 rpm for 5 min, smeared onto microscope slides, air-dried, and stained with Diff-Quik. (**C-4**). Differential cell counts are independently evaluated by two respiratory physicians, each of whom count 500 cells.

BALF handling and processing were conducted in accordance with the guidelines of the ATS and the Japanese Respiratory Society [2,8]. While these protocols were generally followed, the procedures in this study were modified to prioritize simplicity and cost-effectiveness, aiming to enhance feasibility across various healthcare settings as a step toward standardizing BALF processing. BALF samples containing gross mucus were strained through loose gauze when necessary; however, filtration was not performed routinely, as it may alter cellular composition [12]. The first recovered aliquot was used for microbiological and cytological testing, while the second and third aliquots were pooled for further BALF analysis. The pooled samples were divided into five 5 mL aliquots and stored under the following conditions: Group 1–processed within 1 h of collection; Group 2–stored at 4 °C for approximately 6 h; Group 3–stored at room temperature (RT) for approximately 6 h; Group 4–stored at 4 °C for approximately 24 h; and Group 5–stored at RT for approximately 24 h. In contrast to current guideline recommendations [2,8], samples in Groups 2–5 were stored without the addition of a tissue culture medium to reflect practical, real-world conditions and assess the effects of unmodified storage. Total cell counts (TCC) and differential counts of macrophages, lymphocytes, neutrophils, and eosinophils were assessed for each group. After the designated storage period, TCC was measured in duplicate using the TC20^TM^ automated cell counter (Bio-Rad Laboratories Inc., Hercules, CA, USA), and the average value was recorded. The samples were concentrated by centrifugation at 3000 rpm for 5 min, smeared onto microscope slides, air-dried, and stained with Diff-Quick. Cell differentials were independently evaluated by two respiratory physicians (Y.S. and K.M., with 6 and 8 years of experience, respectively), who each counted 500 cells. The final differential counts represent the average of both observers’ assessments. Notably, the evaluation of cell differentials was conducted without blinding to the storage conditions. Figure 1 illustrates the BALF handling, processing, and analysis procedures employed in this study.

### 2.4. Data Collection

The following patient information was collected from medical records: age, sex, smoking history, use of immunosuppressive drugs, and the suspected diagnosis prior to bronchoscopy.

### 2.5. Statistical Analysis

Continuous variables were expressed as medians with ranges. Differences in TCC and cell differentials over time at each temperature condition (4 °C and RT) were analyzed using the nonparametric Kruskal–Wallis test. Due to high inter-patient variability, TCC values were log10-transformed prior to analysis. When the Kruskal–Wallis test yielded significant results, post hoc comparisons were performed using Steel’s test, with the 1 h processing group as the reference. Statistical significance was defined as *p* < 0.05. Additionally, to assess the consistency and reliability of cell differential quantification by two observers, a Gauge Repeatability and Reproducibility (RandR) analysis was conducted. A crossed model was employed to estimate variance components attributable to samples (part-to-part variation), observers (re-producibility), and their interaction (observer part interaction). The Intraclass Correlation Coefficient (ICC), a key metric for evaluating inter-observer agreement, was calculated based on these variance components. All analyzes were conducted using JMP Student Edition software (version 18.2.0; SAS Institute Inc., Cary, NC, USA).

## 3. Results

**Figure 2:**
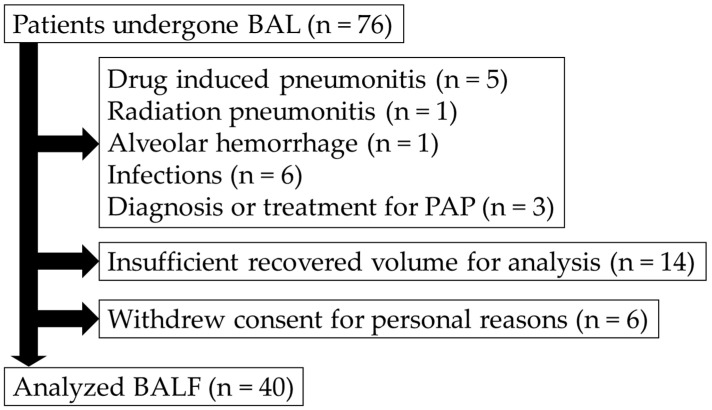
Patient flow diagram. This figure illustrates the details of the patient enrolment and exclusion criteria used in this study. BAL, bronchoalveolar lavage; BALF, bronchoalveolar lavage fluid; PAP pulmonary alveolar proteinosis.

A total of 76 patients were initially enrolled in this study. In total, 6 patients withdrew their consent for personal reasons. A further 16 patients were excluded due to suspected definitive causes of pneumonia, including drug-induced pneumonitis (*n* = 5), radiation-induced pneumonitis (*n* = 1), pulmonary infection (*n* = 6), and pulmonary alveolar proteinosis (*n* = 3), either under treatment or diagnosis. An additional 14 patients were excluded due to insufficient BALF recovery (*n* = 14). In this study, the second and third aliquots of the lavage fluid were used for analysis, requiring a minimum of 25 mL of recovered fluid per case. Furthermore, to accommodate standard clinical procedures such as flow cytometry and to preserve samples for future research, cases deemed to have insufficient recovery by the examiner were excluded from the analysis. The final analysis included data from 40 patients. A detailed flow diagram illustrating patient enrollment and exclusion is shown in Figure 2.

### 3.1. Patient Characteristics

Baseline patient characteristics are summarized in Table 1. The median age was 69 years (range: 31–88 years), with a near-equal sex distribution (18 males and 22 females). Only one participant was a current smoker. Five patients were using immunosuppressive drugs, including inhaled corticosteroids. Pre-bronchoscopy diagnoses included idiopathic interstitial pneumonitis (*n* = 14), connective tissue disease-related ILD (*n* = 9), HP (*n* = 9), sarcoidosis (*n* = 4), pleuroparenchymal fibroelastosis (*n* = 3), and familial interstitial pneumonitis (*n* = 1). BAL was performed in the right middle lobe in 26 patients and in the left lingular lobe in 14 patients. Loose gauze was used for gross mucus removal in five samples. All patients underwent BAL with total of 150 mL normal saline, administered in 50 mL aliquots. The median total recovered volume of BALF was 81.5 mL (range: 48–118) and recovery volume of the 2nd and 3rd aliquots was 65.0 mL (range: 36–85). The median time to analysis was 20 min (range: 6–60 min) for Group 1, 370 min (range: 300–488 min) for Groups 2 and 3, and 1463.5 min (range: 1221–1650 min) for Groups 4 and 5.

### 3.2. TCC

**Figure 3:**
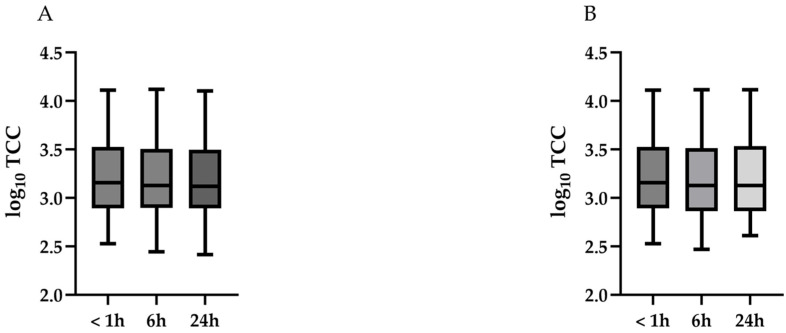
Temporal changes in log10-transformed TCC over time at 4 °C and room temperature (RT). (**A**): No significant changes in log10-transformed TCC were observed over time at 4 °C (*p* = 0.86). (**B**): No significant changes in log10-transformed TCC were observed over time at RT (*p* = 0.90). TCC, total cell count; RT, room temperature.

Table 2 and Figure 3 summarize the changes in log10-transformed TCC over time at 4 °C (Figure 3A) and RT (Figure 3B). No significant changes in log10-transformed TCC were observed over time at either 4 °C (*p* = 0.86) or RT (*p* = 0.90).

### 3.3. Cell Differentials

**Figure 4:**
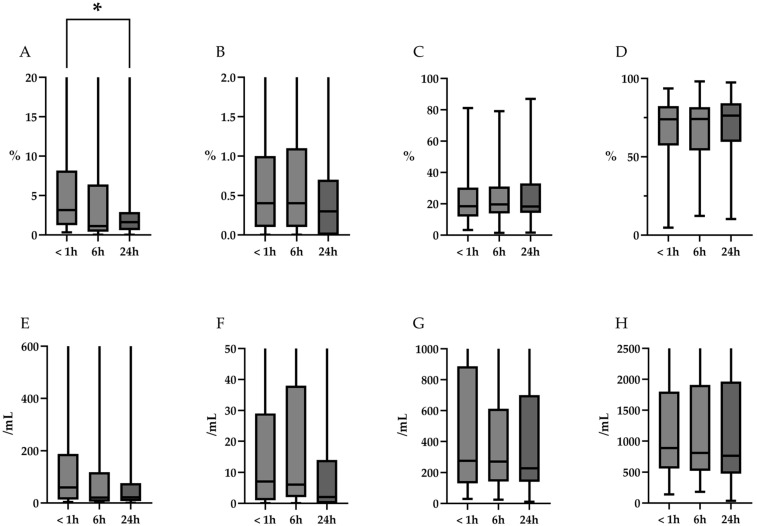
Temporal changes in the proportions and absolute counts of differential cells over time at 4 °C. (**A**–**D**): Changes in the percentages of neutrophils, eosinophils, lymphocytes, and alveolar macrophages, respectively. The percentage of neutrophils decreased significantly over time (*p* = 0.02, indicated by an asterisk (*) in the figure). Post hoc analysis reveals a trend toward reduction at 6 h and a significant decrease at 24 h (*p* = 0.09 and 0.02, respectively). In contrast, the percentages of eosinophils, lymphocytes, and alveolar macrophages remained stable over the 24 h period (*p* = 0.31, 0.79, and 0.74, respectively). (**E**–**H**): Changes in the absolute cell counts calculated based on TCC of neutrophils, eosinophils, lymphocytes, and alveolar macrophages, calculated based on TCC. A decreasing trend was observed for neutrophils over time (*p* = 0.09), whereas the absolute counts of eosinophils, lymphocytes, and alveolar macrophages remained unchanged (*p* = 0.35, 0.94, and 0.90, respectively). TCC, total cell count.

**Figure 5:**
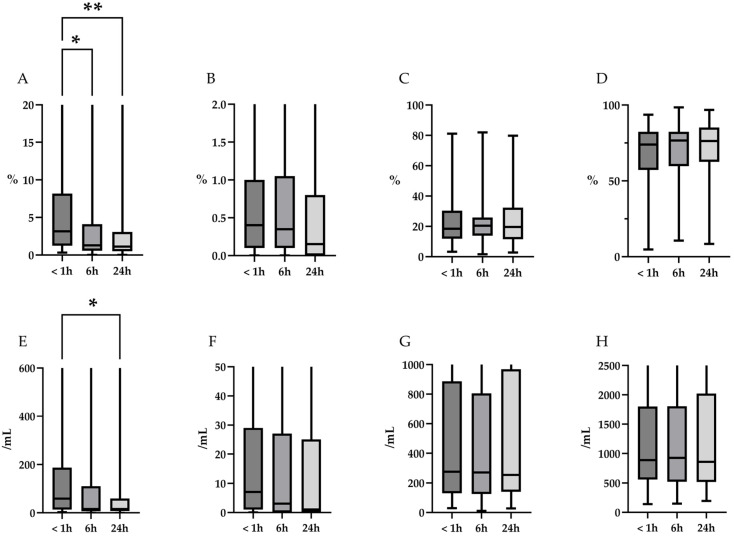
Changes in proportions and absolute counts of differential cells over time at room temperature (RT). (**A**–**D**): Changes in the percentages of neutrophils, eosinophils, lymphocytes, and alveolar macrophages, respectively. The percentage of neutrophils decreased significantly over time (*p* < 0.01). Post hoc analysis revealed significant decreases at 6 and 24 h (*p* = 0.01 and <0.01, respectively; indicated by an asterisk (*) and double asterisk (**), respectively in the figure). In contrast, the percentages of eosinophils, lymphocytes, and alveolar macrophages remained stable over the 24 h period (*p* = 0.32, 0.80, and 0.81, respectively). (**E**–**H**): Changes in the absolute cell counts of neutrophils, eosinophils, lymphocytes, and alveolar macrophages, calculated based on TCC. The absolute counts of neutrophils decreased significantly over time (*p* = 0.03 Indicated by an asterisk (*) in the figure). Post hoc analysis revealed a trend toward reduction at 6 h and a significant decrease at 24 h (*p* = 0.054 and 0.040, respectively). Whereas the absolute counts of eosinophils, lymphocytes, and alveolar macrophages, remained unchanged over time (*p* = 0.38, 0.97, and 0.99, respectively). RT, room temperature; TCC, total cell count.

**Figure 6:**
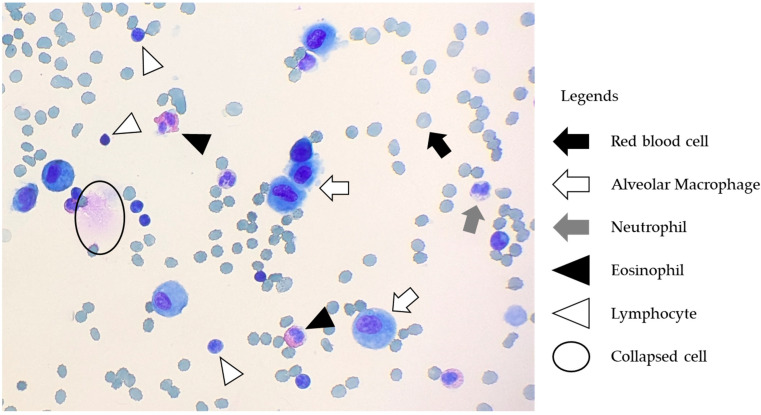
Representative microscopic images. The arrows indicate different cell types, such as red blood cells, alveolar macrophages, neutrophils, eosinophils, and lymphocytes. Circled cells specifically represent collapsed cells.

The changes in cell differentials over time at 4 °C and RT are summarized in Table 3 and Figure 4 (4 °C), and Table 4 and Figure 5 (RT). Representative microscopic images are shown in Figure 6. At 4 °C, the percentage of neutrophils decreased significantly over time (*p* = 0.02). Post hoc analysis revealed a trend toward reduction at 6 h and a significant decrease at 24 h (*p* = 0.09 and 0.02, respectively). In contrast, the percentages of eosinophils, lymphocytes, and alveolar macrophages remained stable over the 24 h period (*p* = 0.31, 0.79, and 0.74, respectively). Furthermore, based on the TCC, a downward trend in absolute neutrophil counts was observed over time (*p* = 0.09), whereas the absolute counts of eosinophils, lymphocytes, and alveolar macrophages remained unchanged (*p* = 0.35, 0.94, and 0.90, respectively). At RT, the percentage of neutrophils also showed a significant decline (*p* < 0.01). Post hoc analysis revealed significant reductions in neutrophil percentage at both 6 and 24 h (*p* = 0.01, and *p* < 0.01, respectively). As observed at 4 °C, the percentages of eosinophils, lymphocytes, and alveolar macrophages remained stable over time (*p* = 0.32, 0.80, and 0.80, respectively). Additionally, the absolute neutrophil counts, calculated based on TCC, decreased significantly over time (*p* = 0.03). Post hoc analysis revealed a trend toward reduction at 6 h and a significant decrease at 24 h (*p* = 0.054 and 0.040, respectively). In contrast, the absolute counts of eosinophils, lymphocytes, and alveolar macrophages, remained unchanged over time (*p* = 0.38, 0.97, and 0.99, respectively).

### 3.4. Inter-Observer Agreement

The ICC results are summarized in Supplement Table S1. While the eosinophil percentage showed relatively low inter-observer agreement (Group 1: 0.13, Group 2: 0.63, Group 3: 0.72, Group 4: 0.65, and Group 5: 0.70, respectively), the ICC values for other cell fractions indicated excellent agreement (ICC > 0.81).

## 4. Discussion

This study evaluated the impact of storage conditions on the TCC and cell differentials in BALF. As hypothesized, delayed processing and storage temperature affected cell differential analyzes, although TCC remained relatively stable over the investigated timeframe. Specifically, the neutrophil percentage significantly decreased over time at both 4 °C and room temperature. Similar changes in neutrophil absolute cell count may reflect true cellular loss.

Although changes in TCC over time have not been validated in human BALF, several studies have examined this in animal models [13,14]. These reports indicate that TCC significantly decreases after 48 h at RT, while remaining stable for up to 72 h at 4 °C. Alveolar macrophages, which constitute the majority of cells in BALF from both healthy individuals and ILD patients [15,16,17,18,19,20], have a reported half-life of 10–30 days in mice [21], likely contributing to the observed TCC stability. Although we did not assess cell viability directly, prior studies have shown that viability is maintained for up to 24 h at 4 °C but decreases significantly within 4–8 h at RT or higher temperatures [9,13]. These findings support current clinical guidelines recommending prompt processing and refrigerated storage of BALF to preserve TCC.

Regarding cell differentials, a previous study involving four patients reported a trend toward reduced percentages of neutrophils, lymphocytes, and eosinophils after 24 h, with more pronounced changes at RT [10]. Similarly, an animal study demonstrated a significant decrease in neutrophils after 24 h at RT and after 48 h at 4 °C. Eosinophil percentages also declined significantly after 24 h at RT [14]. These findings are consistent with our results, particularly the early decline in neutrophils. Given that neutrophil apoptosis begins as early as 9 h after isolation [22,23], the observed decrease is likely attributable to cell degradation over time. Reduced viability may lead to neutrophil lysis and subsequent loss, resulting in a lower proportion of neutrophils detected in the differential count. Eosinophils generally have a lifespan of 2–5 days [24], while the lifespan of lymphocytes remains less well defined. Therefore, differences between previous studies and the present findings may reflect species-specific variations or differences in sample size and experimental conditions.

In the clinical management of ILDs, BALF differential counts, particularly lymphocytes and eosinophils, are used to support diagnostic evaluation and guide anti-inflammatory treatment, including corticosteroid use [5]. In recent years, increasing evidence has suggested that neutrophil percentages in BALF are also prognostically relevant. Elevated neutrophil percentages in BALF have been associated with poor prognosis in idiopathic pulmonary fibrosis (IPF) [3,25,26,27] and progressive fibrosing ILDs [4,28]. Higher neutrophil levels in BALF have also been linked to early mortality in connective tissue disease-associated ILDs [29], and to poorer 90-day survival in patients with acute exacerbation of IPF [30]. Therefore, accurate assessment of neutrophil percentages in BALF is essential, as it may provide prognostic insights. Importantly, our findings indicate that neutrophil percentages are sensitive to both storage time and temperature. Delays in processing or inappropriate storage may result in underestimation of neutrophilic inflammation, potentially affecting clinical interpretation and management.

From a practical standpoint, our findings suggest that for optimal accuracy in cell differential analysis, BALF samples should be processed as soon as possible. If immediate laboratory access is not available, refrigeration at 4 °C can help preserve TCC for up to 24 h; however, even under refrigeration, neutrophil proportions decline significantly within this period. Therefore, if accurate neutrophil counts are essential for clinical decision-making, analysis should ideally occur within a few hours of collection. The importance of standardized BALF handling and storage protocols for improving the diagnostic and prognostic accuracy of ILD cannot be overstated, especially in peripheral centers with limited resources. Establishing uniform guidelines for sample collection, transportation, and processing would reduce pre-analytical variability and significantly enhance the reliability and comparability of BALF analysis across institutions.

This study had several limitations. First, it was a single-center study with a limited sample size. Therefore, future studies conducted at multiple centers with larger cohorts and standardized protocols are needed to assess the external validity of our findings. Second, although all BALF processing and analysis procedures were performed by two physicians, the possibility of variability due to differences in technical skills cannot be excluded. Third, the observers’ assessment of cell fractions may have been influenced by their knowledge of the sample storage conditions, as blinding to these conditions was not implemented during the evaluation. Fourth, storage at room temperature involved fluctuations over time rather than consistent temperature control. Moreover, the study period spanned multiple seasons, further contributing to temperature variability. This lack of stable temperature conditions may have affected changes in cell fractions. Fifth, because BALF was processed and analyzed only at <1, 6, and 24 h, changes that may have occurred between these time points remain unexamined. To better characterize time-dependent changes, future studies should include additional sampling at shorter intervals (e.g., 2, 4, or 8 h). Furthermore, the absence of cell viability assessment, such as trypan blue or 7-AAD staining, may have led to an overestimation of cell differentials. Future research should include cell viability measurements to ensure more accurate and reliable cytological analysis. Finally, the relatively high exclusion rate of approximately 20% due to insufficient recovered volume, although partly attributable to standard clinical specimen processing and alignment with protocols from other studies, may indicate a possible selection bias in our patient cohort.

## 5. Conclusions

Neutrophil percentages in BALF significantly decreased over time, particularly at room temperature, while other cell types remained stable for up to 24 h. These findings highlight the importance of prompt processing or storage at 4 °C to preserve cellular integrity, especially for neutrophils, which are increasingly recognized for their prognostic value in interstitial lung diseases. Standardizing BALF handling protocols could improve diagnostic accuracy and patient outcomes. Future multicenter studies with larger sample sizes and more frequent sampling intervals are warranted to refine optimal storage and processing conditions.

## Figures and Tables

**Table 1:** Baseline characteristics and BAL procedure data.

**Parameter**	**All (*n* = 40)**
Age, years, median (range)	69 (31–88)
Sex (male/female), *n*	18/22
Smoking history (never/past/current), *n*	18/21/1
Usage of immunosuppressive drug, *n*	5
Diagnosis of pre-bronchoscopy test	
IIPs (IPF/NSIP/COP/DIP), *n*	14 (7/4/2/1)
CTD-ILD, *n*	9
HP, *n*	9
Sarcoidosis, *n*	4
PPFE, *n*	3
Familial interstitial pneumonitis, *n*	1
BAL targeted segment, *n*	
right B4	7
right B5	18
right B4+5	1
left B4	8
left B5	5
left B4 + 5	1
Usage of loose gauze for removing gross mucus, *n*	5
Total installed volume, ml (range)	150 (150–150)
Total recovered volume, ml (range)	81.5 (48–118)
Recovered volume of the 2nd and 3rd aliquots, ml (range)	65 (36–85)
Time interval from BAL completion to processing, minutes (range)	
Group 1	20 (6–60)
Groups 2 and 3	370 (300–488)
Groups 4 and 5	1463.5 (1221–1650)

COP, cryptogenic organizing pneumonia; CTD-ILD, connective tissue disease-related interstitial lung disease; DIP, desquamative interstitial pneumonia; HP, hypersensitivity pneumonia; IIPs, idiopathic interstitial pneumonitis; IPF, idiopathic pulmonary fibrosis; NSIP, nonspecific interstitial pneumonitis; PPFE, pleuroparenchymal fibroelastosis.

**Table 2:** Temporal changes in log_10_-transformed TCC over time at 4 °C and RT.

Parameter		<1 h	6 h	24 h	*p* Value
4 °C	log_10_ TCC, median (range)	3.16 (2.54–4.11)	3.13 (2.44–4.12)	3.12 (2.41–4.10)	0.86
RT	log_10_ TCC, median (range)	3.16 (2.54–4.11)	3.13 (2.47–4.11)	3.13 (2.61–4.12)	0.90

TCC, total cell count; RT, room temperature.

**Table 3:** Changes in percentage/proportion of cell differentials over time at 4 °C.

Parameter	<1 h	6 h	24 h	*p* Value
Neutrophils, % median (range)	3.3 (0.3–92.0)	1.1 (0.0–82.5)	1.6 (0.0–81.8)	**0.02**
Post hoc Steel test, 6 h/24 h	-	-	-	0.09/**0.02**
Cell counts of neutrophils, /mL, median (range)	59 (3–6385)	20 (0–5247)	22 (0–3751)	0.09
Eosinophils, % median (range)	0.4 (0.0–5.7)	0.4 (0.0–8.9)	0.3 (0.0–8.5)	0.31
Cell counts of eosinophils, /mL, median (range)	7 (0–180)	6 (0–269)	2 (0–139)	0.35
Lymphocytes, %, median (range)	18.4 (3.2–81.0)	19.6 (1.4–79.1)	18.2 (1.6–86.9)	0.79
Cell counts of lymphocytes, /mL, median (range)	276 (29–6102)	270 (24–6351)	228 (11–7416)	0.94
Alveolar macrophages (AM), %, median (range)	73.9 (4.8–93.6)	74.1 (12.3–98.0)	76.2 (10.3–97.4)	0.74
Cell counts of AM, /mL, median (range)	890 (139–9765)	812 (182–8705)	765 (34–9634)	0.90

Statistically significant values (*p* < 0.05) are shown in bold in the table.

**Table 4:** Temporal changes in proportions of differential cells over time at RT.

Parameter	<1 h	6 h	24 h	*p* Value
Neutrophils, % median (range)	3.3 (0.3–92.0)	1.3 (0.0–81.8)	1.1 (0.0–81.6)	**<0.01**
Post hoc Steel test, 6 h/24 h	-	-	-	**0.01/<0.01**
Cell counts of neutrophils, /mL, median (range)	59 (3–6385)	16 (0–4986)	15 (0–4957)	**0.03**
Post hoc Steel test, 6 h/24 h	-	-	-	0.054/**0.04**
Eosinophils, % median (range)	0.4 (0.0–5.7)	0.4 (0.0–6.4)	0.2 (0.0–5.5)	0.32
Cell counts of eosinophils, /mL, median (range)	7 (0–180)	3 (0–209)	25 (0–161)	0.38
Lymphocytes, %, median (range)	18.4 (3.2–81.0)	20.4 (1.6–81.9)	19.7 (2.7–79.8)	0.80
Cell counts of lymphocytes, /mL, median (range)	276 (29–6102)	270 (11–6708)	255 (27–7282)	0.97
Alveolar macrophages (AM), %, median (range)	73.9 (4.8–93.6)	76.6 (10.5–98.4)	76.3 (8.4–96.8)	0.81
Cell counts of AM, /mL, median (range)	890 (139–9765)	928 (147–10777)	862 (193–4798)	0.99

Statistically significant values (*p* < 0.05) are shown in bold in the table. RT, room temperature.

## Data Availability

The data presented in this study are available upon request from the corresponding author.

## Supplementary Materials

The following supporting information can be downloaded at https://www.mdpi.com/article/10.3390/diagnostics15111386/s1, Supplement Table S1. The Inter-observer agreement evaluated by the Intraclass Correlation Coefficient.

## Abbreviations

The following abbreviations are used in this manuscript:

ATS	American Thoracic Society
BAL	Bronchoalveolar lavage
BALF	Bronchoalveolar lavage fluid
HRCT	High-resolution computed tomography
ICC	Intraclass Correlation Coefficient
ILD	Interstitial lung disease
RT	Room temperature
TCC	Total cell counts
