# Supplementary material for: Impact of Storage Conditions on Bronchoalveolar Lavage Fluid Analysis: A Human Study
Source: Diagnostics (Basel). 2025 May 30;15(11):1386. doi: 10.3390/diagnostics15111386 (PMC12154212; doi:10.3390/diagnostics15111386)
Supplement: Supplementary file 1 [file diagnostics-15-01386-s001.zip › diagnostics-3616797-supplementary.pdf]

Supplement Table S1. The Inter-observer agreement evaluated by the Intraclass Correlation Coefficient

	**Neutrophil**	**Eosinophil**	**Lymphocyte**	**Alveolar Macrophage**
**Group 1**	0.96	0.13	0.90	0.87
**Group 2**	0.97	0.62	0.92	0.94
**Group 3**	0.98	0.72	0.91	0.92
**Group 4**	0.98	0.65	0.91	0.93
**Group 5**	0.96	0.70	0.92	0.92
